# Atg5-Independent Sequestration of Ubiquitinated Mycobacteria

**DOI:** 10.1371/journal.ppat.1000430

**Published:** 2009-05-15

**Authors:** Cathleen A. Collins, Ann De Mazière, Suzanne van Dijk, Fredric Carlsson, Judith Klumperman, Eric J. Brown

**Affiliations:** 1 Department of Microbial Pathogenesis, Genentech, Inc., South San Francisco, California, United States of America; 2 Biomedical Sciences Graduate Program and Medical Scientist Training Program, University of California San Francisco, San Francisco, California, United States of America; 3 Cell Microscopy Center, Department of Cell Biology and Institute for Biomembranes, University Medical Center Utrecht, Utrecht, The Netherlands; University of Washington, United States of America

## Abstract

Like several other intracellular pathogens, *Mycobacterium marinum* (Mm) escapes from phagosomes into the host cytosol where it can polymerize actin, leading to motility that promotes spread to neighboring cells. However, only ∼25% of internalized Mm form actin tails, and the fate of the remaining bacteria has been unknown. Here we show that cytosolic access results in a new and intricate host pathogen interaction: host macrophages ubiquitinate Mm, while Mm shed their ubiquitinated cell walls. Phagosomal escape and ubiquitination of Mm occured rapidly, prior to 3.5 hours post infection; at the same time, ubiquitinated Mm cell wall material mixed with host-derived dense membrane networks appeared in close proximity to cytosolic bacteria, suggesting cell wall shedding and association with remnants of the lysed phagosome. At 24 hours post-infection, Mm that polymerized actin were not ubiquitinated, whereas ubiquitinated Mm were found within LAMP-1–positive vacuoles resembling lysosomes. Though double membranes were observed which sequestered Mm away from the cytosol, targeting of Mm to the LAMP-1–positive vacuoles was independent of classical autophagy, as demonstrated by absence of LC3 association and by Atg5-independence of their formation. Further, ubiquitination and LAMP-1 association did not occur with mutant avirulent Mm lacking ESX-1 (type VII) secretion, which fail to escape the primary phagosome; apart from its function in phagosome escape, ESX-1 was not directly required for Mm ubiquitination in macrophages or *in vitro*. These data suggest that virulent Mm follow two distinct paths in the cytosol of infected host cells: bacterial ubiquitination is followed by sequestration into lysosome-like organelles via an autophagy-independent pathway, while cell wall shedding may allow escape from this fate to permit continued residence in the cytosol and formation of actin tails.

## Author Summary

*M. tuberculosis* is one of the world's most prevalent pathogens, infecting one third of humans and contributing to 2 million deaths each year. *M. marinum* (Mm) has been increasingly studied as a model of *M. tuberculosis* due to its relative safety and its shared mechanisms of pathogenesis; for example, previous studies have highlighted the importance of the secretion system ESX-1 in pathogenesis of both *M. tuberculosis* and Mm. Here, we show that the host's ubiquitin system, best known for tagging host proteins for degradation, also recognizes Mm that have escaped their original phagosomes via an ESX-1–dependent mechanism and entered into the cytosol. Ubiquitinated bacteria have two distinct fates; ubiquitin tagged Mm can be sequestered into lysosome-like compartments, but they also can shed cell wall, which may allow them to evade sequestration. Lysosomal sequestration is independent of autophagy, a response to starvation or infection that leads to degradation of organelles and certain other intracytosolic pathogens. These experiments reveal ubiquitination as a mechanism of host cell recognition of mycobacteria and the existence of a new interface between the host and invading microbe that may be amenable to therapeutic intervention.

## Introduction

*Mycobacterium marinum* (Mm) is a close genetic relative of the important human pathogen *M. tuberculosis*, and shares with *M. tuberculosis* the ability to infect host macrophages, as well as to induce a similar pathologic response [1],[2]. As in mammalian models of infection with *M. tuberculosis*, loss of the ESX-1 secretion system greatly attenuates the virulence of Mm in its natural fish and amphibian hosts [3]–[5]. While the reason that ESX-1 is required for *M. tuberculosis* virulence remains unknown, there is some understanding of its role in pathogenesis of Mm infections. After Mm infection of host cells, ESX-1 is required for bacterial escape from the phagosome, recruitment of uninfected macrophages, and subsequent cell-to-cell spread [6]–[8]. Although whether or not *M. tuberculosis* ever escapes from phagosomes remains highly controversial [9],[10], loss of ESX-1 activity in this organism also leads to failure of cell-to-cell spread [11].

Some Mm polymerize actin, forming actin tails after entering the host macrophage's cytosol [12]. However, this represents only a fraction (∼25%) of the intracellular bacilli; the fate of the remaining 75% of infecting bacteria has yet to be established. To explore the fate of intracellular Mm, we developed an assay that accurately distinguishes intraphagosomal from cytosolic bacteria. Surprisingly, bacteria escaped from phagosomes to the cytosol many hours before actin polymerization was evident. A proportion of these newly-escaped cytosolic bacteria were ubiquitinated, as was bacteria-derived material that appeared to be shed from the mycobacterial surface and incorporated into host membranous structures.

Distantly related pathogens which can escape the phagosome, *Salmonella typhimurium* and *Listeria monocytogenes*, also associate with ubiquitin, but whether this represents direct ubiquitination of the bacterial surface or close apposition of ubiquitinated host proteins remains unproven, and the significance of the association is unclear [13]. Similar to ubiquitin associated *Listeria*, ubiquitinated Mm did not form actin tails; rather, they became associated with LAMP-1 positive macrophage lysosomes, suggesting a reuptake process from the cytosol.

The process of autophagy, which engulfs organelles and proteins into characteristic double membrane vacuoles during cell stresses including starvation and formation of intracytosolic protein aggregates [14],[15], is a mechanism for host defense against some intracellular pathogens escape primary phagosomes, including *Salmonella*, *Listeria*, *Streptococcus pyogenes*, and *Shigella flexneri* [16]–[19]. For each of these organisms, autophagic host defense is dependent on Atg5, a central component in the formation of autophagic membranes. Autophagy may also be important in host defense against Mycobacteria. Enhancement of autophagy in macrophages by starvation or rapamycin treatment leads to association of autophagy markers with *M. tuberculosis* var. *bovis* bacilli Calmette Guérin (BCG) phagosomes and overcomes the block in phagosome-lysosome fusion characteristic of infection with BCG and *M. tuberculosis* [20]. However, sequestration of ubiquitinated Mm in LAMP-1 positive compartments did not require induction of autophagy and was independent of Atg5, suggesting an alternative pathway for reuptake of these organisms from the cytosol. These studies demonstrate that Mm undergo a complex kinetic interaction within the macrophages that are their preferred replication niche. After escape from phagosomes, some bacteria polymerize actin to facilitate cell-to-cell spread, while others become ubiquitinated, leading to reuptake into a host vesicular compartment by a mechanism distinguishable from classic autophagy. These alternative fates may contribute to the wide variety of effects of ESX-1 on host-pathogen interactions during mycobacterial infection [21]–[23].

## Results

### *M. marinum* escape from phagosomes hours before activation of actin polymerization

**Figure 1:**
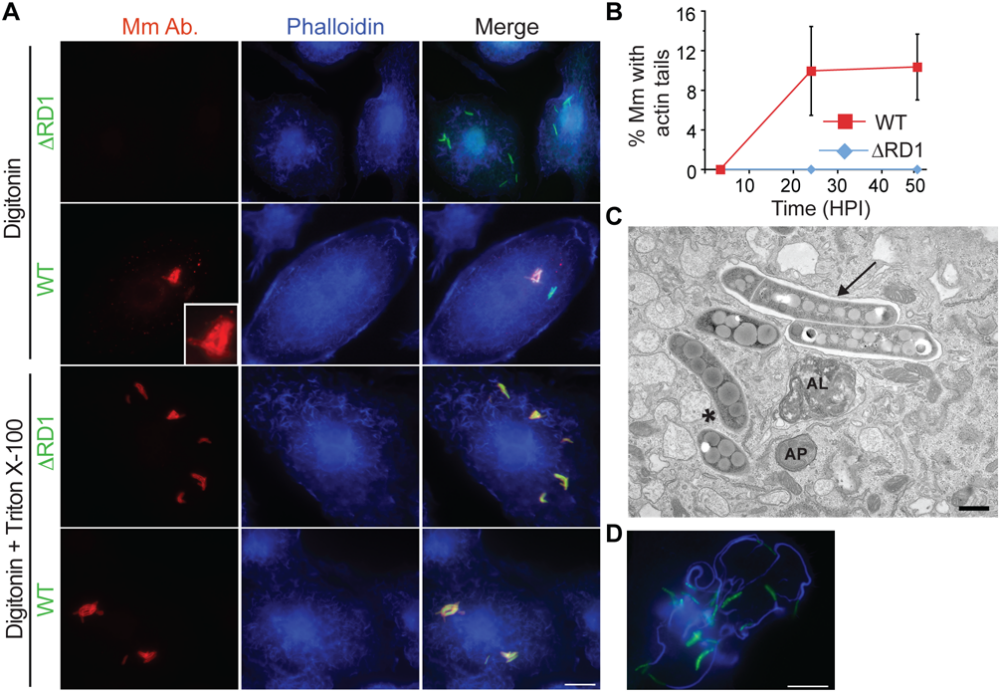
Mm escapes from phagosome by 3.5 HPI. (A) 3.5 HPI with ΔRD1 (1st and 3rd rows) or WT (2^nd^ and 4^th^ rows) Mm expressing GFP, macrophages were permeabilized with digitonin alone (top two rows) or digitonin followed by TX-100 (3^rd^ and 4^th^ row) as described in Materials and Methods. Bacteria were stained with an anti-Mm antibody (1^st^ column, red) and polymerized actin was visualized with phalloidin-Alexa 350 (2^nd^ column, blue). Third column is a merge of the images in the first two columns. Inset in the first panel of the second row shows a 3.5 fold magnification of the cytosolic WT Mm in the larger panel, stained with the Mm antibody. (Scale bar, 10 µm). (B) Actin tails, identified by phalloidin-Alexa 350 after digitonin and TX-100 staining, were quantified by counting all bacteria from at least 50 infected macrophages for WT (squares, red line) and ΔRD1 (diamonds, blue line) at various times after infection in three independent experiments and graphed as mean±SD. (C) Epon section of a portion of a macrophage infected with WT Mm at 3.5 HPI. An asterisk denotes a Mm in the cytosol, an arrow points to Mm enclosed in phagosome membrane. AP, autophagosome; AL, autolysosome. Scale bar, 500 nm. (D) A macrophage infected with WT Mm expressing GFP 24 HPI stained for phalloidin-Alexa 350 (blue) as in (A) (Scale bar, 10 µm). This image shows Mm exhibiting robust actin motility within a single cell, and is shown to illustrate several examples of actin polymerization at 24 HPI; it is not representative of the average 10% polymerizing actin in the entire population of infecting Mm as quantitated in (B).

We found that digitonin could permeabilize macrophage plasma membranes, while leaving phagosome membranes intact, as previously described [24] (Figure S1A). We combined macrophage permeabilization with digitonin with an antibody to Mm cell walls to detect cytosolic mycobacteria. A proportion of wildtype (WT) Mm in infected macrophages could be stained after digitonin permeabilization, whereas Mm lacking region of difference 1 (ΔRD1), which have no functioning ESX-1 secretion system, failed to be stained by antibody to the bacterial cell wall under these conditions (Figure 1A, top two rows). This was not due to failure of the antibody to recognize the mutant bacteria, since all intracellular ΔRD1 Mm were stained after macrophage permeabilization with Triton X-100, which solubilized phagosome as well as plasma membranes (Figure 1A, third row). Thus, lack of staining after digitonin permeabilization was indicative of the presence of ΔRD1 Mm in phagosomes. All WT Mm were also stained with antibody under the same conditions of Triton X-100 permeabilization (Figure 1A, bottom row). Using this technique, we found that ∼21% of WT Mm had escaped from phagosomes by 3.5 hours post infection (HPI), a time before significant bacterial actin polymerization occurred, as detected by phalloidin staining (Figure 1A and 1B). Bacterial escape from phagosomes also was demonstrated by electron microscopy, which showed about 43% of WT Mm in the cytosol at 3.5 HPI, while 100% of ΔRD1 Mm were clearly enclosed within a membrane (Figure 1C, Figure S1B, S1C). As previously described [12], actin polymerization by WT Mm was not detected at 3.5 HPI and increased dramatically by 24 HPI (Figure 1B and 1D). Thus, WT Mm escape from macrophage phagosomes several hours prior to initiation of actin-based motility.

### Ubiquitination of cytosolic *M. marinum*

**Figure 2:**
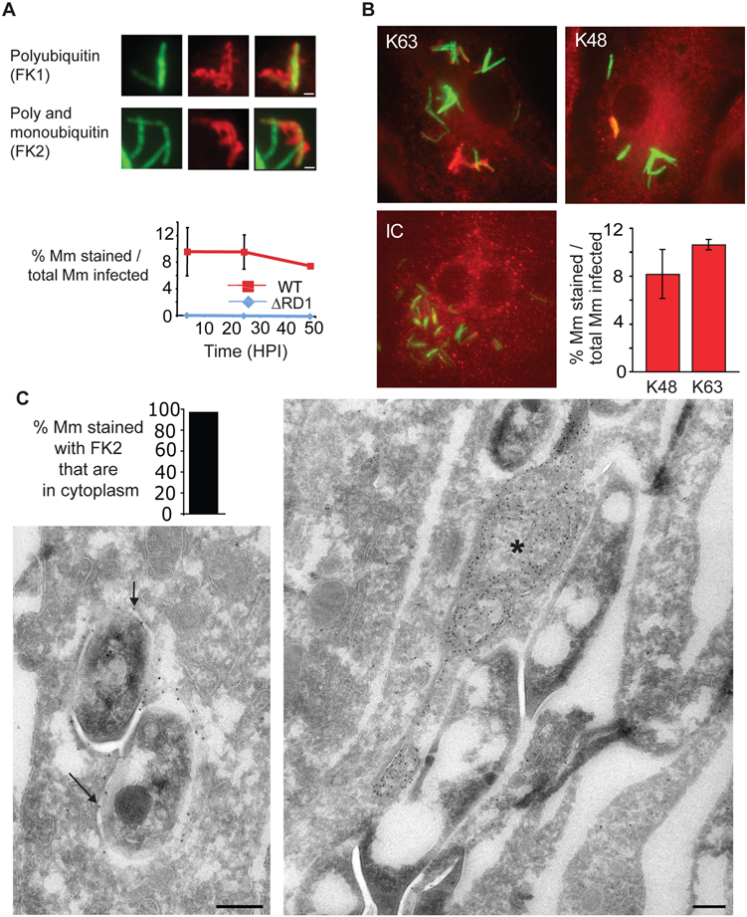
Cytosolic Mm associates with polyubiquitin. (A) 48 HPI with WT Mm expressing GFP, macrophages were stained with antibodies for polyubiquitin (FK1) or poly- and monoubiquitin (FK2). Left panel, GFP fluorescence; middle panel, anti-ubiquitin fluorescence; right panel is a merge of the two other panels, showing co-localization of both anti-ubiquitin antibodies with Mm. (Scale bars, 1 µm). Graph shows quantification of percent FK2 staining of WT (squares, red line) and ΔRD1 (diamonds, blue line), Mm from at least 50 infected macrophages at each time point in three independent experiments and graphed as mean±SD. (B) 24 HPI post infection, macrophages were stained with monoclonal antibodies recognizing K63-linked polyubiquitin (left panel), K48-linked polyubiquitin (middle panel), or anti-her2 as an isotype control (IC). Specificity of linkage detection was demonstrated by competition with K48 or K63 tetraubiquitin chains (Figure S2A). Graph shows quantification of percent of intracellular WT Mm stained with K63- and K48- polyubiquitin linkage-specific antibodies in macrophages infected for 24 hours. The difference in K63 and K48 association with Mm were not statistically significant (P = 0.0662), determined as described in Materials and Methods. Data are from at least 50 infected macrophages at each time point in each of three independent experiments and graphed as mean±SD. (C) Ultrathin cryosection of 3.5 HPI of macrophages with WT Mm, labeled with FK2 anti-ubiquitin, and 10 nm protein-A gold particles. Left panel shows Mm with ubiquitin on bacterial surface, indicated with arrows. Right panel shows ubiquitin on membranous structures associated with Mm, labeled with an asterisk. Graph shows quantification percent of ubiquitin associated Mm which were cytosolic or intra-vacuolar. 48 out of 50 such organisms were cytosolic. Scale bars, 200 nm.

Because of the long delay between phagosome escape and initiation of actin-based motility, we hypothesized that cytosolic Mm might be recognized by ubiquitinating enzymes, similar to other bacterial species that escape the phagosome [13]. Polyubiquitin was found associated with WT Mm, since bacteria were stained by an antibody recognizing only polyubiquitin (FK1), as well as an antibody that recognizes monoubiquitin-derivatized surfaces as well as polyubiquitin chains (FK2) (Figure 2A). Approximately 9% of WT Mm taken up by macrophages stained with FK2 at all times from 3.5 to 48 h (Figure 2A). Comparison of this percentage with the 21% escape observed at 3.5 HPI suggests that about 40% of all bacteria in the cytosol associate with polyubiquitin at this time.

Often, the cell biologic effects of polyubiquitin are determined by whether the ubiquitin chains are assembled through K48 or K63 linkages. While K48 polyubiquitin is most often a signal for proteasomal or lysosomal degradation, K63 linkages have been associated with assembly of signaling scaffolds [25]. By using antibodies specific for K48 and K63 poyubiquitin linkages [26], we found that both chain types associated with WT Mm. Though K48-linked polyubiquitin association with Mm was more variable than K63, the proportions were not statistically distinct (Figure 2B). Specificity of the linkages was demonstrated by competition with K48 or K63 tetraubiquitin chains (Figure S2A).

To determine whether the ubiquitinated Mm were cytosolic or intraphagosomal, infected macrophages were permeabilized with digitonin and stained with both anti-ubiquitin and antibody to the Mm cell wall, which under these conditions recognizes cytosolic but not intraphagosomal bacteria (Figure 1A). This procedure revealed that virtually all ubiquitinated WT Mm were cytosolic at 3.5 HPI, as WT Mm that bound FK2 also were stained by the Mm cell wall antibody (Figure S2B). Quantitative immunoelectron (EM) microscopy confirmed the cytosolic localization of ubiquitinated WT Mm at 3.5 HPI, as 96% of ubiquitin associated WT Mm were in the cytosol (Figure 2C, left image and graph). In addition to direct association with intact bacteria, ubiquitin was frequently found associated with adjacent dense membrane structures (Figure 2C, right image), which appeared as networks with meshes and broadened parts, containing electron-dense material and occasionally small vesicles (and are designated further in the text as dense membrane networks).

**Figure 3:**
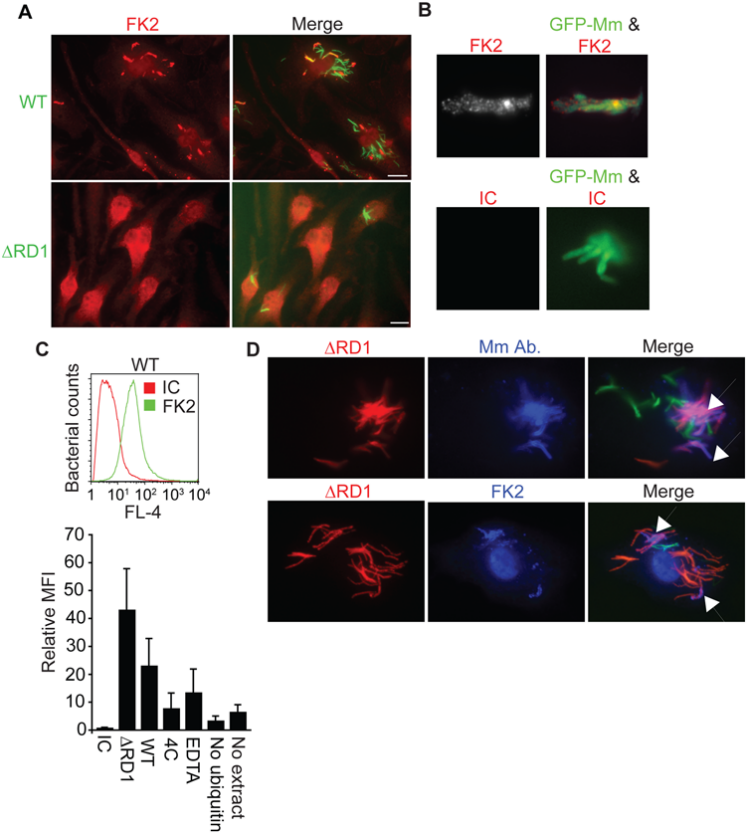
ESX-1 is necessary for Mm ubiquitination in macrophages. (A) Macrophages were infected with WT (top panels) or ΔRD1 (bottom panels) Mm and stained with the FK2 antibody to poly- and monoubiquitin at 24 HPI (Scale bar, 10 µm). (B) Mm were ubiquitinated *in vitro* by incubating Mm with HeLa cell cytosolic extracts for 2 hours at 36°C as described in Materials and Methods. Polyubiquitination of bacteria was detected by immunofluorescence after staining with FK2; isotype control is an antibody to HIV gp120. (C) Flow cytometry was performed to quantify ubiquitination of Mm. Fluorescence of Mm incubated with HeLa cell extracts and stained as in (B) was measured using FACSCaliber. The histogram plot shows a representative log scale distribution of the number of bacteria (y axis) vs. fluorescence intensity for mAb staining of Mm (x axis) stained with antibody to poly- and monoubiquitin (FK2, green histogram) or with isotype control anti-gp120 antibody (IC, red histogram). The bar graph shows the ratio of mean fluorescent intensity of FK2 staining for each condition to ubiquitinated WT Mm stained with the isotype control antibody. Data expressed as mean±SD from three independent experiments. (D) Coinfection of macrophages with WT and ΔRD1 Mm. Macrophages were coinfected with WT Mm expressing GFP and ΔRD1 Mm expressing RFP at a ratio of 1∶1. 24 HPI macrophages were permeabilized with digitonin and stained with anti-Mm (top row, middle panel) to detect cytosolic bacteria, or with FK2 after permeabilization with Triton X-100 to detect bacterial ubiquitination (bottom row, middle panel). Merged images in the right panels show WT-GFP (green), ΔRD1-RFP Mm (red) and anti-Mm (blue in top panel) or FK2 (blue in bottom panel). Arrows point to ΔRD1-RFP Mm stained by anti-Mm in cytosol (top right) or ubiquitinated (bottom right). In absence of coinfection, ΔRD1 Mm were never seen ubiquitinated or found in cytosol (data not shown).

These results suggested that WT Mm ubiquitination occurred upon direct contact of the bacteria with macrophage cytosol, a hypothesis supported by the lack of ubiquitin association with ΔRD1 Mm, which never become cytosolic (Figure 2A, Figure 3A, data not shown). In contrast, the attenuated *iipA* mutant, which escapes phagosomes but grows poorly in macrophages [27], did become polyubiquitinated, suggesting that lack of ubiquitin association with ΔRD1 Mm did not reflect simple loss of virulence or decreased intracellular growth (Figure S3A).

To determine whether defects in ΔRD1 Mm besides failure to escape phagosomes affected its ubiquitination, HeLa cell cytosol was incubated with WT and ΔRD1 Mm, as we found HeLa cells also ubiquitinated Mm during infection (data not shown). Deposited ubiquitin was detected with FK2. Immunofluorescence showed ubiquitin localization in bright patches on the surface of WT Mm, with little background staining from an isotype control antibody (Figure 3B). Quantification of ubiquitin deposition was done by flow cytometry (Figure 3C, top panel), which showed that bacterial ubiquitination was abrogated in the absence of HeLa cytosol, after chelation of divalent cations to remove Mg-ATP, which is required for activity of the E1 ubiquitin activating enzyme, and at 4°C (Figure 3C, bottom). Binding of ubiquitin to WT Mm likely was covalent, as washing multiple times with 8 M urea to disrupt noncovalent interactions did not inhibit staining with FK2 (Figure S3B). In this *in vitro* assay, ΔRD1 was ubiquitinated equivalently or at greater levels than WT, suggesting that the lack of ubiquitin association with ΔRD1 Mm during infection in macrophages was due to lack of phagosome escape rather than absence of the substrate for ubiquitination.

To test this hypothesis directly, we coinfected macrophages with ΔRD1 and WT Mm at a ratio of 1∶1. Under conditions of coinfection, ΔRD1 Mm escaped from phagosomes, as shown by staining of ΔRD1-RFP with Mm cell wall antibody after permeabilization with digitonin alone (Figure 3D, top panel), presumably because of phagosome lysis by WT. Under conditions of coinfection, ΔRD1 also became polyubiquitinated (Figure 3D, bottom panel). These results demonstrate that both *in vitro* and in cells, ΔRD1 Mm can be ubiquitinated when exposed to host cytosol. Thus, the failure of ΔRD1 to be ubiquitinated in monoinfection (i.e., when used alone to infect macrophages) results from its failure to escape from phagosomes.

### Ubiquitin-associated Mm are incorporated into intracellular LAMP-1–positive compartments independent of autophagy

**Figure 4:**
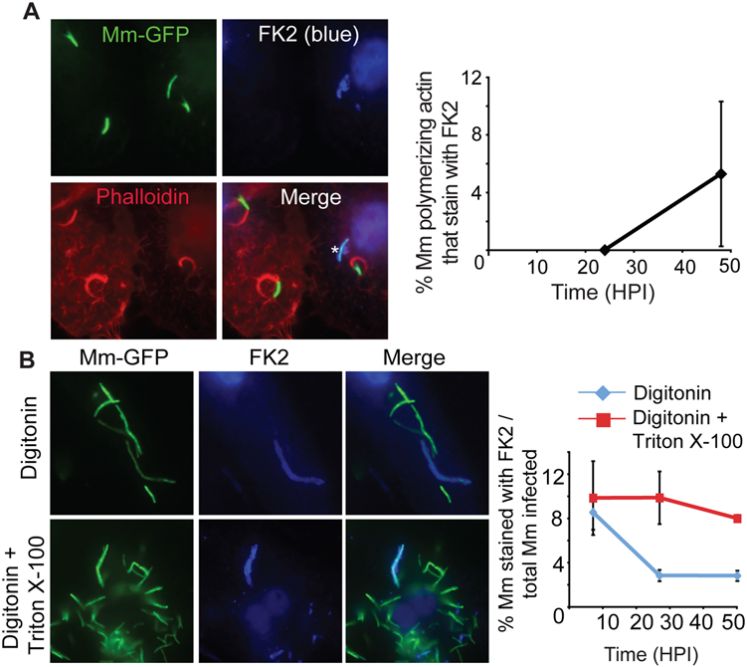
Sequestration of ubiquitinated Mm. (A) 24 HPI with WT Mm expressing GFP, macrophages were permeabilized with Triton X-100 and stained with FK2 (blue, top right) and phalloidin-Alexa 594 (red, bottom left). In merged image (bottom right), ubiquitinated Mm (shown with asterisk) had no association with polymerized actin. The graph on the right shows the percentage of Mm with actin tails that also stain with FK2 quantified 24 and 48 HPI in fifty infected macrophages. Data shown as the mean±SD of three independent experiments. (B) 24 HPI with WT Mm, macrophages were permeabilized with digitonin (top row) or digitonin followed by Triton X-100 (bottom row) and subsequently stained with FK2 (blue). Merged images of the two panels is shown in the right panels. Maintenance of phagosome integrity after digitonin permeabilization was confirmed by lack of staining of ΔRD1 with the Mm antibody (data not shown). The graph on the right shows the percentage of ubiquitinated Mm at various times after infection in macrophages permeabilized with digitonin alone (diamonds, blue line) or with digitonin followed by Triton X-100 (squares, red line). Data are mean±SD of three independent experiments, each examining at least 50 infected macrophages at each time point.

**Figure 5:**
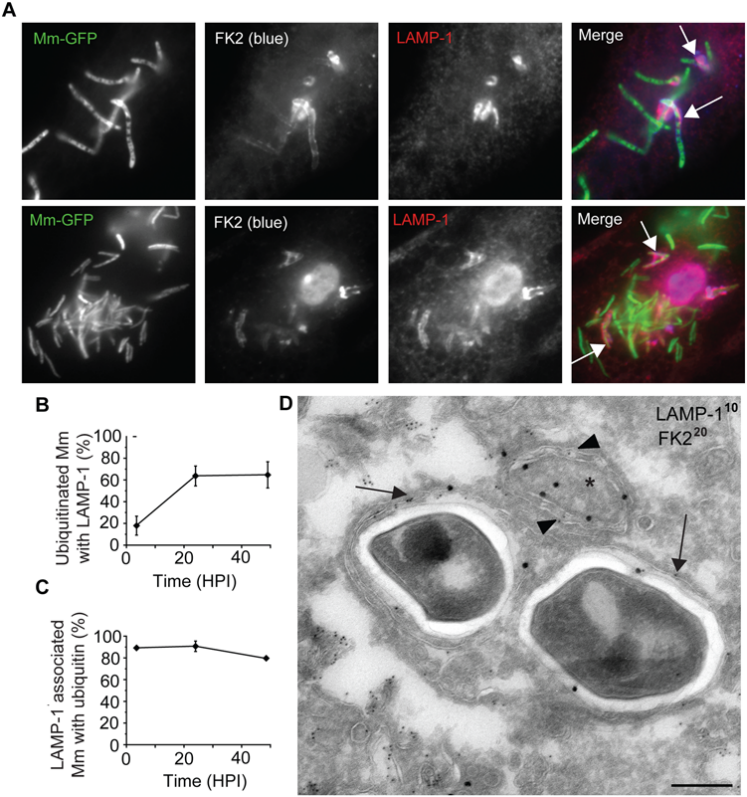
Association of ubiquitinated Mm with LAMP-1. (A) 48 HPI macrophages infected with GFP-expressing WT Mm were fixed and permeabilized with Triton X-100 and subsequently stained with FK2 (2^nd^ panel, blue) and antibody to LAMP-1 (3^rd^ panel, red). Merged images of the green, red, and blue fluorescence is shown in the right panel. Arrows point to bacteria showing co-localization of ubiquitin and LAMP-1, with top row showing LAMP-1 in discrete patches and bottom row showing ubiquitinated bacteria surrounded by LAMP-1. (B) Quantification of percent of ubiquitinated Mm that co-localize with LAMP-1 at various times after macrophage infection. Data are depicted as mean±SD of three independent experiments, with at least 50 macrophages counted per experiment. (C) Quantification of percent LAMP-1 positive Mm that ubiquitinated at various times after macrophage infection. Data are depicted as mean±SD of three independent experiments, with at least 50 macrophages counted per experiment. (D) Ultrathin cryosections of macrophages 24 HPI with WT Mm were double-labeled with anti–LAMP-1 (10 nm gold particles) and FK2 anti-ubiquitin (20 nm gold particles). Arrows point to examples of LAMP-1 labeling on membranes surrounding ubiquitinated Mm. Immediately adjacent to the Mm, ubiquitinated material (asterisk) is enclosed within a double membrane positive for LAMP-1 (arrowheads). Scale bar, 200 nm.

Despite the association of ubiquitin with cytosolic bacteria at 3.5 HPI, we did not observe any ubiquitin-associated WT Mm that formed actin tails at 24 HPI (Figure 4A). Instead, at this time the ubiquitinated bacteria appeared to be reinternalized into membrane bound compartments, because the frequency of detection of ubiquitinated bacteria stained after digitonin permeabilization decreased, even though the total number of ubiquitinated bacteria detected after Triton X-100 permeabilization did not change over time (Figure 4B). At 24 and 48 HPI, fewer than 20 to 25% of ubiquitinated WT Mm were detected after plasma membrane permeabilization with digitonin alone. In support of the hypothesis that lack of accessibility to FK2 represented resequestration into a membrane-bound compartment, ubiquitinated WT co-localized with the late endosomal/lysosomal marker LAMP-1 (Figure 5A). Some Mm appeared with LAMP-1 in discrete patches (top row of Figure 5A) while the entire contour of others appeared to be surrounded with LAMP-1 (bottom row of Figure 5A); these patterns suggested recruitment and fusion of lysosomes to surround ubiquitinated Mm. By 24 HPI, 64% of ubiquitinated bacteria were associated with LAMP-1, a frequency that did not change at later times (Figure 5B). At all time points, almost all bacteria associated with LAMP-1 were ubiquitinated (Figure 5C). In addition, in three independent experiments, at 3.5 and 24 HPI, respectively, 5.7%±6.15% (SD) and 0% of ΔRD1 Mm co-localized with LAMP-1 when macrophages were infected with these ESX-1–deficient bacteria alone, implying that WT Mm in LAMP-1 positive compartments were derived from those that had escaped into the macrophage cytosol. To further characterize the nature of the LAMP-1 positive compartments, we next studied these at the ultrastructural level, using immunoEM. LAMP-1 positive membranes surrounded FK2 positive WT Mm and FK2 positive material close to the Mm (Figure 5D). The FK2 positive material was surrounded by two membranes enclosing a narrow, electron-lucent space, as in the case of an autophagosome, but containing LAMP-1, which is unusual for autophagosomal membranes (Figure 5D).

**Figure 6:**
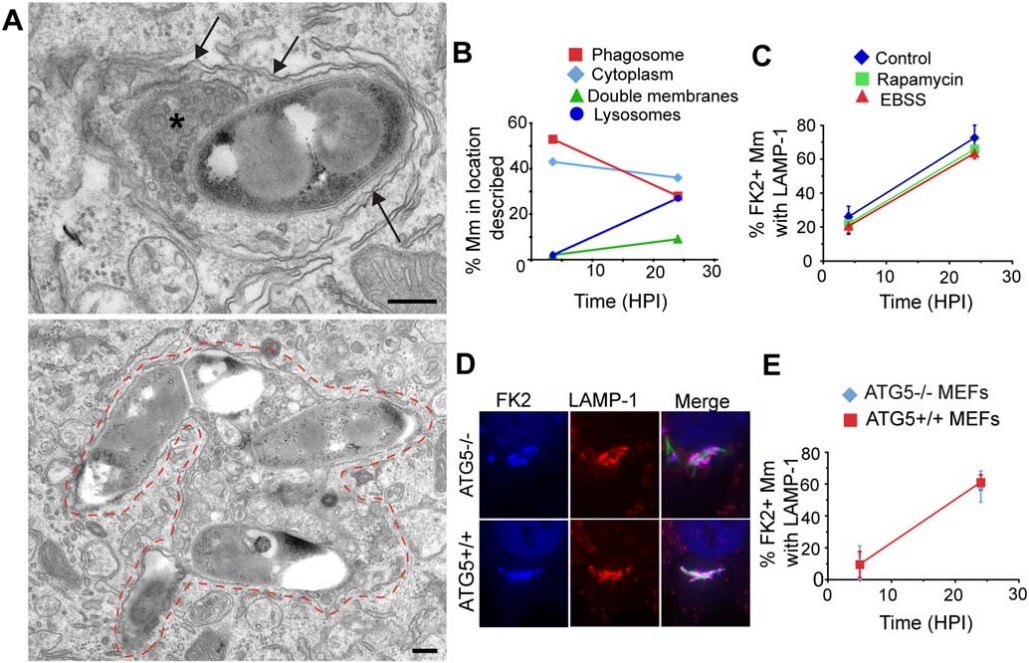
Atg5-independence of Mm sequestration. (A) At 24 HPI, many Mm appear enclosed in membrane-lined compartments morphologically similar to autophagosomes and autolysosomes. The top image shows a bacterium in close association with a double membrane structure suggestive of an autophagosome (arrows). The double membranes also appear to extend around characteristic dense material (asterisk), similar to the ubiquitinated material shown adjacent to Mm in Figure 2C (right image). The bottom image shows Mm enclosed in an single membrane (auto)lysosomal vacuole containing a large amount of membranes and debris in addition to the bacteria, with dashes showing the outline of the vacuole. Scale bars, 200 nm. (B) Quantification of number of Mm in single membrane phagosomes (squares, red line), the cytosol with no associated membranes (diamonds, light blue line), in double membrane structures (triangles, green line) or single membrane lysosomes (circles, dark blue line) exemplified in Figure 6A. The 3.5 HPI data is from one experiment (N = 177) and the 24 HPI data is the average of two independent experiments (N = 138 and 160). (C) Autophagy was induced in macrophages by preincubating cells with rapamycin (squares, green line), or EBSS (triangles, red line) for 2 hours before infection. Control cells were incubated in standard macrophage growth media (diamonds, blue line). 4 or 24 HPI of these cells, the percentage of ubiquitinated bacteria co-localizing with LAMP-1 was determined as described in Figure 5. The graph shows the mean±SD of 50 cells per condition at each time point for three separate experiments. (D) ATG5−/− (top row) and ATG5+/+ (bottom row) MEFs were infected with GFP-expressing WT Mm for 24 hours and stained for ubiquitin (left panels, blue in merged image) and LAMP-1 (middle panels, red in merged image). As in macrophage infection, ubiquitinated Mm co-localizing with LAMP-1 are evident. (E) The percentage of ubiquitinated Mm co-localizing with LAMP-1 was determined at 5 and 24 HPI. Graph represents mean±SD of three independent experiments for ATG5+/+ MEFs (squares, red line) or ATG 5−/− MEFS (diamonds, blue line). At least 50 infected cells were counted at each time point in each experiment.

The morphology of the LAMP-1 positive compartment containing ubiquitinated WT Mm was difficult to define by immunoEM, as it was compromised by the pre-embedding labeling procedure, including permeabilization, required for FK2 labeling (see Materials and Methods). Therefore, to examine in more detail the nature of the membrane compartments enclosing the Mm, electron microscopic analysis was performed on cells directly embedded (without permeabilization) in Epon resin at various times after macrophage infection. At 3.5 HPI, 43% WT Mm appeared to be in the cytosol and 53% in phagosomes. 2% appeared in double membrane vacuoles, consistent in morphology with autophagosomes (Figure 6A, top panel), and 2% were in lysosomes containing heterogeneous material including organelles and membranes (Figure 6A, bottom panel). By 24 HPI the number of WT Mm in vesicles with the morphologic characteristics of autophagosomes and lysosomes increased to 9% and 27%, respectively (Figure 6A and 6B). 100% of ΔRD1 appeared to be in phagosomes at 3.5 and 24 HPI (N = 83 and 19 respectively for each time point, data not shown).

Because the double membrane structures into which ubiquitinated WT Mm were sequestered resemble autophagosomes, and *M. tuberculosis* has been reported to localize to autophagosomes and then to autolysosomes after induction of autophagy [20], we tested whether LC3, an early marker for autophagy [14], co-localized with ubiquitinated WT Mm at various times after infection. While LC3 occasionally appeared closely apposed to both FK2 stained and unstained WT Mm in both control and starved cells, (Figure S4A), fewer than 3% of ubiquitinated WT Mm co-localized with LC3 at 4 and 24 hours, and overall less than 1% of WT associated with LC3 (Figure S4C). As positive controls for LC3 staining, anti-LC3 detected LC3 aggregates in macrophages after induction of autophagy by starvation or after rapamycin treatment, associated with a conversion of LC3-I to the membrane bound form LC3-II, (Figure S4B, S4F). Thus, there was insignificant association of LC3 with ubiquitinated WT Mm at any point examined during macrophage infection.

To determine whether induction of autophagy could enhance association of ubiquitinated WT Mm with LAMP-1, macrophages were starved or treated with rapamycin 2 hours before infection, and co-localization of ubiquitinated bacteria with LAMP-1 positive vesicles was determined 4 and 24 HPI. Though LC3-I was converted to LC3-II under these conditions (Figure S4F), neither starvation nor rapamycin increased the rate of uptake of ubiquitinated WT into a LAMP-1 positive compartment (Figure 6C). Preincubation of macrophages with IFN-γ, which induces autophagy in the RAW264.7 macrophage cell line (20), also did not affect Mm targeting to LAMP-1 positive vacuoles (Figure S4D). In the absence of additional stimulation to autophagy, there was no increased conversion of LC3-I to LC3-II in infected macrophages between 6 and 24 HPI, while resequestration of cytosolic WT Mm was occurring, over the minimal levels in uninfected controls (Figure S4E), suggesting that bacterial infection does not induce an autophagy response under these conditions. Thus, our experiments provide no evidence that the sequestration of WT Mm into LAMP-1 positive compartments depends on conversion of LC3 or its association with Mm-containing vesicles.

These results raised the possibility that an autophagy-independent pathway led to the sequestration of ubiquitinated WT Mm into LAMP-1 positive membrane-bound vesicles. To test definitively whether autophagy was necessary for the targeting of ubiquitin-associated WT Mm to LAMP-1 positive vacuoles, we assessed infection of Atg5^−/−^ mouse embryo fibroblasts, since Atg5 is a required component of the pathway for autophagosome formation [28]. As previously shown, Atg5^−/−^ MEFs were unable to convert LC3-I to LC3-II after starvation and were thus incapable of activating the autophagy pathway (Figure S4G). During infection of MEFs, WT Mm escaped from phagosomes and polymerized actin [29], were ubiquitinated (Figure 6D), and ubiquitinated WT Mm were sequestered into LAMP-1 positive compartments (Figure 6E), thus recapitulating the major events in Mm infection of macrophages. Moreover, ubiquitinated mycobacteria associated with LAMP-1 in Atg5^+/+^ and Atg5^−/−^ MEFs with similar kinetics, increasing over time from about 10% at 5 hours to about 60% at 24 hours (Figure 6D and 6E). Thus, the incorporation of cytosolic ubiquitinated WT Mm into a LAMP-1 positive compartment occurred independently of the events of classical autophagy. In addition, lack of Atg5 had little effect on the survival and replication of Mm at 24 HPI, with infected Atg5^+/+^ MEFs having 1.03±0.32 fold the colony forming units as Atg5^−/−^ MEFs (average±SEM of three independent experiments).

### Mycobacterial cell wall alteration during infection

**Figure 7:**
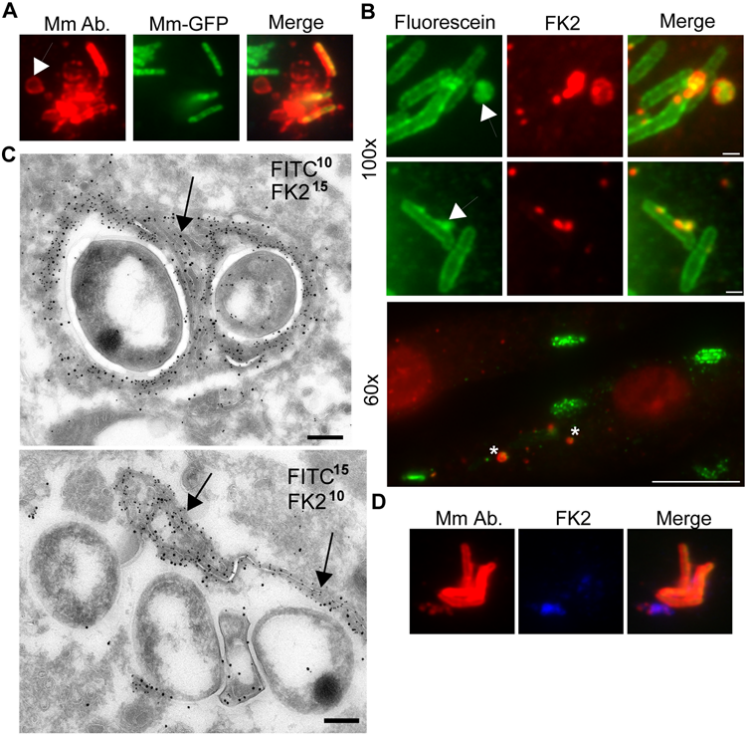
Release of ubiquitinated Mm surface molecules in macrophage cytosol. (A) Macrophages infected for 3.5 hours with GFP-expressing Mm were permeabilized with digitonin and stained with anti-Mm antibody (left panel, red). Middle panel shows GFP fluorescence and right panel is a merged image of the green and red fluorescence. Arrow points to a region of Mm antibody staining distinct from intact bacteria. (B) Mm were labeled with CFSE prior to infection of macrophages, which were subsequently stained with FK2 anti-ubiquitin. CFSE fluorescence is shown in the left panel (green), and FK2 staining in the middle panel (red). Top two rows show 100× magnification of cells fixed at 6.5 HPI (Scale bar, 1 µm), demonstrating release of CFSE labeled, ubiquitinated material (arrows); bottom image shows a 60× magnification demonstrating multiple collections of released, ubiquitinated Mm material at 3.5 HPI (asterisks) (Scale bar, 10 µm). (C) Mm were labeled with CFSE as above for immunoelectron microscopy. Ultrathin cryosections of macrophages at 3.5 HPI were double-labeled with FK2 and anti-fluorescein, each followed by protein-A gold particles of the indicated sizes in nm. Both images of co-localization at the bacterial surface (upper image) and co-localization on the characteristic membranes (arrows) associated with the bacteria (upper and lower image) were obtained. Note that in the lower image, there is little staining of the bacterial surface with either anti- fluorescein or anti- ubiquitin. Scale bars, 200 nm. (D) Macrophages were infected with GFP-expressing Mm, stained with anti-Mm after digitonin permeabilization (left panel, red) and FK2 (middle panel, blue). Merge shows co-localization of anti-Mm with FK2 staining.

Our EM images suggested that, in addition to the ubiquitin attached directly to the mycobacterial cell wall, there was a large amount of ubiquitin associated with dense membrane networks in close proximity to the cytosolic bacteria (Figure 2C, right image). These typical membrane networks were only seen upon macrophage infection with WT Mm and not in uninfected cells or during infection by ΔRD1 Mm (data not shown). We found that these membranes were at least in part Mm-derived because when infected macrophages were permeabilized with digitonin and stained with anti-Mm cell wall antibodies, bacterial cell wall material was often localized in small dots or vesicles near cytosolic bacteria by immunofluorescence (Figure 7A). Moreover, when bacteria were fluoresceinated prior to macrophage infection, following methods developed previously [30], shed cell wall material could be seen at 3.5 HPI both by electron microscopy using an anti-fluorescein antibody and by direct fluorescence microscopy (Figure 7B and 7C). This shed cell wall material was often ubiquitinated, and appeared to be incorporated in the dense membrane networks adjacent to cytosolic bacteria (Figure 7B–7D). Host membranes also contributed to this “peri-bacterial” ubiquitinated material: when cholera toxin B was used to label the macrophage lipid raft marker GM1 for 8 minutes before infection, it co-localized with these ubiquitinated membranes at 3.5 hours after infection (Figure S5). In addition, in immunoEM preparations, the fluorescein- and ubiquitin-positive membranes had the appearance of a bilayer and not of an Mm cell wall (Figure 2C, Figure 7C). These host- and Mm- derived dense membrane networks may represent residual phagosome membranes, with which mycobacterial cell wall molecules remain associated after Mm escape. Alternatively, released hydrophobic Mm cell wall molecules in the cytosol may mix nonspecifically with host membranes. Thus, it appears that Mm shed ubiquitinated cell wall material in macrophages. Since we observed only ubiquitinated mycobacteria in LAMP-1 positive vacuoles, shedding of the cell wall may represent a strategy to evade this fate. This view is supported by the EM observation of the dense membrane networks enclosed in a double membrane lining (data not shown).

**Figure 8:**
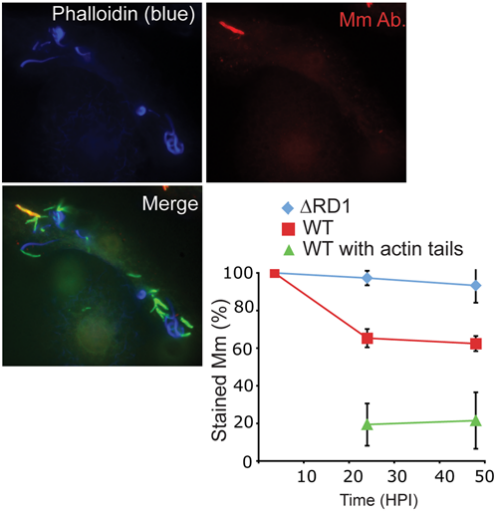
Alteration in Mm antigenicity during macrophage infection. 24 HPI with GFP-expressing WT Mm, macrophages were permeabilized with digitonin and Triton X-100 and stained with phalloidin-Alexa 350 (left panel, blue in bottom merged image) and anti-Mm (right panel, red in bottom merged image). Multiple cytosolic bacteria that polymerized actin failed to be recognized by anti-Mm. In bottom right panel, the percentage of infecting Mm that stained with the anti-Mm antibody was quantitated for ΔRD1 (diamonds, blue line) and WT Mm (squares, red line) during 48 h of infection. The percentage of WT with actin tails that stained with the anti-Mm antibody (triangles, green line) also was determined. Graph shows mean±SD for three independent experiments.

To test whether any alterations in Mm cell wall antigenicity might accompany the shedding of ubiquitinated cell wall antigens, Mm were stained with anti-cell wall antibody at various times after infection, using TX-100 permeabilization to allow the antibody access to all intracellular bacteria. 100% of both WT and ΔRD1 Mm stained with the antibody at 3.5 hours, and ΔRD1 continued to be recognized by the cell wall antibody throughout the 48 h experiment (Figure 8, quantified in bottom right panel). In contrast, at 24 and 48 HPI, only about 60% of WT Mm stained with the antibody. The changes to the cell wall did not appear to be a direct mechanism of ubiquitin evasion, since at 24 and 48 hours only 71% and 28% of ubiquitinated WT Mm stained with the antibody, indicating that Mm which exhibited changes to their cell wall could still be ubiquitinated (data not shown). The ubiquitination of Mm exhibiting cell wall changes hints at a continuous process, since Mm exhibit these changes only at later time points of infection and thus must be ubiquitinated after many hours of residence within the macrophage, possibly after shedding their initial wall. In contrast to ubiquitin association, actin polymerization and staining with the anti-cell wall antibody were negatively correlated, since 20% of WT Mm with actin tails at 24 and 48 HPI stained with the Mm antibody (Figure 8, quantified in bottom right panel). This result suggests that cell wall alterations during residence in the cytosol may be required for WT Mm to gain the ability to polymerize actin.

## Discussion

**Figure 9:**
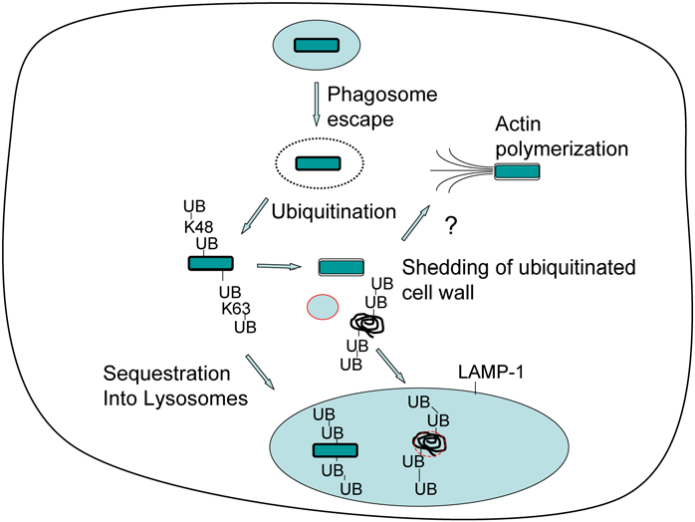
Alternative fates for cytosolic Mm during macrophage infection. After uptake into a macrophage phagosome Mm (green bar) escapes quickly into the cytosol. Cytosolic Mm may be ubiquitinated, leading to resequestration of bacteria into a LAMP-1–positive vesicle. Alternatively, ubiquitinated bacteria may shed ubiquitin with components of the bacterial cell wall. Unubiquitinated bacteria polymerize actin.

Escape from phagosomes underlies the mechanism by which Mm and distantly related bacteria such as *Shigella* and *Listeria* enhance cell-to-cell spread of infection [31]. For Mm, phagosome escape requires a specialized secretion system, ESX-1 [6]. Cytosolic bacteria polymerize actin to induce motility, the mechanism underlying the increased cell-to-cell spread. Cytosolic bacterial products also can activate innate immunity through sensors such as NODs [32]. In this study we describe an entirely different fate for a subset of cytosolic bacteria. These bacteria become ubiquitinated and then are resegregated from the cytosol into a LAMP-1–positive compartment, which forms independently of Atg5, an essential component of classical autophagosome formation. Our data indicate that Mm may have evolved strategies to avoid sequestration, apparently involving cell wall shedding to deplete the ubiquitin signal and alterations in cell wall antigenicity that may promote actin polymerization, as illustrated in the model in Figure 9.

The observation that cytosolic bacteria were either ubiquitinated or motile (with actin tails), but not both, suggests that these are alternative fates. Since both actin polymerization and ubiquitination are self-reinforcing, there may be competition between the two reactions at the bacterial surface, with either outcome negatively feeding back on the other. If this is the case, the outcomes of actin polymerization or ubiquitination may be determined by specific characteristics of the bacteria's surface during residence in the cytosol.

Previous studies had determined that Mm do not gain the ability to polymerize actin until many hours after initial infection and had inferred from this fact that escape from phagosomes was likewise slow [12]. In the current study, we have developed methods to examine phagosome escape independent of actin polymerization and show, both by differential solubilization techniques and by electron microscopy, that bacteria enter the cytosol much earlier than previously thought. By 3.5 HPI, 20–40% of internalized bacteria have escaped from phagosomes to the cytosol. Early escape allows access of the mycobacterial wall to ubiquitination machinery; because we observed that a large amount of ubiquitinated cell wall was lost from the bacteria, Mm may continuously shed ubiquitinated wall molecules into the cytosol, allowing time for the underlying deubiquitinated surface to express surface characteristics required for actin polymerization (Figure 9). The ubiquitination of Mm with significant cell wall alterations, as demonstrated by loss of reactivity with antibody to *in vitro* grown bacteria, hints at an ongoing process, since these Mm must be ubiquitinated after this antigenic shift, possibly after shedding their original wall.

Ubiquitinated Mm were sequestered in LAMP-1 positive vacuoles. We considered the possibility that the membranes recapturing cytosolic Mm were generated through autophagy, as this process has been identified as an important pathway in innate defense against several intracellular pathogens, including both Gram positive or (*L. monocytogenes* and *S. pyogenes*) and Gram negative (*S. typhimurium* and *S. flexneri*) bacteria as well as *M. tuberculosis* [17],[18],[16],[19],[20]. However, our evidence suggests that resegregation of Mm from the cytosol by the LAMP-1–positive macrophage membranes is by a process distinct from classical autophagy, since the majority of bacteria-associated host membranes do not express LC3, a marker of autophagosomal membranes, and their formation is independent of Atg5. This is quite distinct from the other bacterial pathogens known to be incorporated into cytosolic vesicles, which have clearly associated LC3-containing membranes dependent on Atg5 for their formation. These results also differ from studies highlighting the importance of autophagy in targeting *M. tuberculosis* var. *bovis* bacilli Calmette Guérin (BCG) to lysosomes, since autophagy induction enhances BCG co-localization with lysosomal markers LAMP-1, cathepsin D, LBPA and the V_o_H^+^-ATPase [20]. Autophagy induction also decreased survival of BCG and *M. tuberculosis* H37Rv, while we observed no difference in Mm's survival in wild type or Atg5 deficient MEFs [20]. It is possible that the differences we observe between Mm and BCG are due to the latter bacteria's propensity to remain in the primary phagosome, while Mm clearly has an intracellular phase when it is not enclosed in a membrane-bound vacuole. BCG appears not to activate autophagy as a direct consequence of infection, but to become entrapped in an autophagosome after induction by another signal or after IFN-γ activation of macrophages [20],[33]. In contrast, ubiquitinated Mm become resequestered in lysosome-like vacuoles without any additional extrinsic signal. Possibly, autophagy engulfs BCG because of a damaged phagosomal membrane, similar to the autophagic clearance of *Salmonella typhimurium* in response to perforation of the *Salmonella* containing vacuole (SCV) rather than full escape of the microbe [16]. However, ubiquitination and non-autophagic sequestration may occur later during infection with *M. tuberculosis*, since there is evidence that after two days it escapes to the cytosol of human monocyte-derived dendritic cells and macrophages through an ESX-1 dependent mechanism [10].

We speculate that ubiquitination is required for Mm uptake into LAMP-1 positive compartments, since virtually all bacteria associated with LAMP-1 are ubiquitinated, but this hypothesis remains unproven. Our evidence suggests that the segregation of Mm that have escaped into the cytosol occurs by a process of membrane formation initiated by a biochemical pathway distinct from classical, Atg5 dependent autophagy. One possibility is that the reincorporation of ubiquitinated Mm into a membranous compartment intersects with the ESCRT pathway, which delivers ubiquitinated cargo to multivesicular bodies and finally to lysosomes [34]. The double membranes could point to elongated or cup-shaped lysosomes as have been described in liver parenchymal cells [35]. In addition, the function of these compartments needs to be further established. Clearly, the LAMP-1 positive membranes segregate Mm away from the cytosol, but whether they also act as degradative compartments remains to be established.

Ubiquitination is a signal for uptake of aggregated proteins into autophagosomes, a process that requires the ubiquitin binding protein p62 [36]. At this time it is not clear why ubiquitinated Mm do not trigger this autophagy response. In this regard, it is interesting that both *Listeria* and *Shigella* actively inhibit entrapment by autophagy; *Listeria* uses actin polymerizing ActA as well as other proteins to evade autophagy, while *Shigella* secretes IcsB, resulting in disruption of the interaction between Atg5 and VirG, another actin polymerizing protein [17],[19]. Our results showing no effect of deficiency of Atg5 on Mm growth are similar to experiments with wild type *Listeria* [17]. The low level of autophagy of Mm we observed, as well as its similar ability to polymerize actin, suggests the possibility of autophagy inhibition by Mm.

Ubiquitination of Mm may also have other functions, such as targeting of the bacteria or ubiquitinated membranes to the proteasome. Ubiquitinated *Salmonella* seems to be degraded by the proteasome, while *Listeria* is not [13]. While we observed little association of the 20 s proteasome with ubiquitinated Mm at 24 and 48 HPI, attempts to study involvement of the proteasome were limited since inhibition of proteasome activity with MG-132 led to loss of ubiquitination of Mm, most likely through depletion of free ubiquitin (data not shown). Regardless of whether the proteasome can degrade whole bacteria, its degradation of ubiquitinated bacterial membranes may be important in presentation of bacterial antigens via the MHC Class I pathway, a prominent feature of Mycobacterial infection [37],[38].

An growing list of bacterial effectors have been shown to interfere with the host's ubiquitination system, acting as ubiquitin ligases to target host proteins for degradation, dampening signaling through inflammatory pathways such as NF-κB [39]. However, the reciprocal bacterial surface molecules recognized by the host ubiquitination machinery have yet to be identified. Our results expand the list of known bacteria that are targeted by ubiquitin, as both Gram-positive *Listeria monocytogenes* and the Gram-negative *Salmonella typhimurium* associate with polyubiquitin during infection as discussed above. The mycobacterial cell wall has many distinct features, including a thick mycolic acid layer which distinguishes it from those of conventional Gram-positive and Gram-negative bacteria [40]. In addition, our *in vitro* ubiquitination experiments constitute the first evidence that bacterial association with ubiquitin is a true covalent association with the bacterial surface. A prior study demonstrated that ubiquitin can come into contact with *Mycobacterium tuberculosis* and BCG in the lysosome, where the ubiquitin peptides are toxic to the bacteria [41], but in the present study several lines of evidence demonstrate that Mm ubiquitination occurs in the cytosol. Most significantly, at early times after infection the vast majority of ubiquitin-associated Mm are in the cytosol, as judged both by their exposure to antibodies after digitonin permeabilization and by electron microscopy. In addition, the ΔRD1 mutant is never ubiquitinated when infecting macrophages alone, but can be ubiquitinated when allowed to come in contact with cytosol by coinfection with WT Mm, or by mixing with host cell cytosol. This demonstrates that ESX-1 is not required for expression of the targets of ubiquitination, but is necessary for exposure of Mm to cytosolic ubiquitinating enzymes. Ubiquitination may be a response of the host to bacterial cell wall proteins or other molecules that it detects as abnormal in some way, similar to the response to unfolded or modified proteins. The large number of mycobacterial cell wall lipopeptides and proteins with high proline content or hydrophobic sequences [42] are likely candidates for recognition by the host ubiquitination machinery [43]. However, the presence of K63-linked chains on the bacteria, usually associated with signal transduction pathways rather than protein degradation, suggests that marking bacterial proteins for destruction is not the only consequence of Mm ubiquitination [25].

In summary, Mm exhibits two apparently distinct fates upon prolonged residence in the cytosol of infected macrophages (Figure 9). On one path, ubiquitinated bacteria are sequestered into LAMP-1 positive compartments; this may limit spread of infection and lead to direct toxicity to the enclosed bacteria; on the other path, bacteria shed cell wall material and possibly escape ubiquitination and sequestration. This may permit further growth as well as dramatic changes to the mycobacterial cell wall, actin polymerization and spread of infection. We speculate that both fates may influence the outcome of infection, since sequestration may enhance antigen presentation and activate or modulate pathways for cytokine production. At the same time, these data hint at a novel pathway for delivery of cytosolic material into lysosomes, independent of classical autophagy. It will be important and interesting to determine the mechanisms involved and whether these processes are shared by other pathogenic mycobacteria, as well as other microbes that share Mm's life style of phagosome escape and actin polymerization.

## Materials and Methods

### Antibodies and reagents

Antibodies to *M. marinum* (Mm) were generated by injecting rabbits with cell wall fractions from ΔRD1 Mm grown in Sauton's media. Phalloidin-Alexa 350 and phalloidin-Alexa 594 were from Invitrogen (Carlsbad, CA). Mouse monoclonal anti-conjugated ubiquitin antibodies FK1 and FK2 were from Biomol (Plymouth Meeting, PA). K48, K63, anti-her2, and anti-HIV gp120 antibodies were produced at Genentech (South San Francisco, CA). The ID4B rat monoclonal LAMP-1 antibody was from BD Biosciences (San Jose, CA) and the LC3 antibody was from Novus Biologicals (Littleton, CO). The fluorescein antibody was from Zymed Laboratories (South San Francisco, CA). The GAPDH antibody was from Millipore (Temecula, CA). Cholera toxin B-Alexa 594 was from Invitrogen (Carlsbad, CA). Secondary antibodies used for EM were rabbit anti-mouse IgG and anti-rat IgG (Dako, Glostrup, Denmark).

### Bacteria and cell culture

WT Mm (strain M) and ΔRD1 Mm [44] were cultured in Middlebrook 7H9 (BD, Franklin Lakes, NJ) supplemented with 0.2% glycerol/0.05% Tween 80/10% ADC Enrichment (Fisher Scientific, Pittsburgh, PA). Bone marrow derived macrophages were harvested from 129/SvJ mice, cultured as previously described, and used between day 7 and 21 of growth [45]. Wild type and Atg5−/− MEFs, a kind gift of Noboru Mizushima, were maintained in DMEM containing 10% FBS.

### Infections

2×10^5^ macrophages or 1×10^5^ MEFs were plated overnight in DMEM containing 10%FBS, 20 mM Hepes, 10% CMG14-12 supernatant [46] or DMEM with 10% FBS, respectively. 1 hour before infection, cells were washed with PBS and placed in DMEM containing 3% FBS and 3% CMG for macrophages or 3% FBS for MEFs. Mm were washed twice in PBS and passaged through a 26-gauge needle three times to disrupt aggregates. Mm were added to macrophages at MOI of 4 to 12 for 2 hours at 32°C, and macrophage monolayers then were incubated with 200 µg/ml amikacin for 2 hours, following PBS wash, to kill extracellular organisms. After a final PBS wash, macrophages were incubated in DMEM containing 3% FBS & 3% CMG14-12 supernatant at 32°C in a 5% CO_2_ humidified incubator. Alternatively, MEFs were infected for 3 hours at an MOI of 40, washed and incubated with 200 µg/ml amikacin for 2 hours, washed to remove the drug and incubated for the indicated time. To count colony forming units, MEFs were infected for 5 hours, washed with PBS and lysed with 0.25% Triton X-100 in PBS after 24 hours.

### Differential permeabilization with digitonin

Infected macrophages were washed 2× with KHM buffer (110 mM potassium acetate, 20 mM Hepes and 2 mM MgCl_2_), incubated with KHM containing 25 ug/mL digitonin for 1 minute at room temperature and then washed 1× with detergent-free KHM buffer. Macrophages were then incubated with antibody (anti-Mm and/or FK2) in KHM containing 2% BSA or with buffer alone at 32°C for 12 minutes, washed with PBS and fixed with 4% PFA. Macrophages that had been incubated with buffer alone were then permeabilized with 0.2% Triton X-100 for 4 minutes and then stained with the appropriate primary antibodies, or the appropriate secondary antibodies if they had already received the primary. Bound antibody was visualized with Alexa 350-conjugated goat anti- mouse F(ab')_2_ (Invitrogen, Carlsbad, CA) for FK2 or Alexa 594-conjugated goat anti- rabbit F(ab')_2_ (Invitrogen, Carlsbad, CA) by incubating in 2% BSA in PBS for 30 minutes. Controls omitting primary antibodies showed minimal fluorescence from the secondary antibodies alone, minimal crossreactivity of the anti-mouse F(ab')_2_ or anti- rabbit F(ab')_2_ with bound antibody of the other species, and insignificant bleedthrough of green fluorescence into the red channel and vice versa. Stained samples were viewed using an Axioplan 2 light microscope (Carl Zeiss MicroImaging) with a Semrock filter set and images were recorded with a CoolSNAP_HQ_ CCD camera (Photometrics).

### Immunoelectron microscopy

Macrophages were fixed with 2% PFA and 0.2% glutaraldehyde in 0.1 M phosphate buffer, pH 7.4 for 2 hours at room temperature. Alternatively, they were fixed for 12 minutes with 4% PFA, permeabilized with 0.1% saponin for 6 minutes, incubated with FK2 or isotype control anti-gp120 antibody for 45 minutes, washed with PBS and postfixed with 2% PFA and 0.2% GA for 2 hours. After rinsing with PBS, the blocks were embedded in 12% gelatin, cryoprotected with 2.3 M sucrose, and frozen in liquid nitrogen as previously described [47]. Ultrathin cryosections were cut at −120°C, picked up with 1% methylcellulose, 1.2 M sucrose, thawed and collected on copper grids. After washing with PBS containing 0.02 M glycine, sections were incubated with rabbit anti mouse IgG followed by protein-A gold for the detection of FK2 in permeabilized cells, or single- or double-labeled with primary (and secondary) antibodies followed by protein-A gold as described earlier [47]. The sections were then contrasted with a 1.8% methylcellulose, 0.6% uranyl acetate mixture. Quantification of Mm ubiquitination was performed on minimally 200 randomly screened WT Mm from each time point (3.5 HPI, 24 HPI), from at least 2 experiments.

### Electron microscopy

Cells were fixed either with 2% PFA and 0.2% glutaraldehyde in 0.1 M phosphate buffer, or with half-strength Karnovsky fixative (2% paraformaldehyde, 2.5% glutaraldehyde, and 0.1 M sodium cacodylate buffer) pH 7.4 for 2 hours at room temperature. After rinsing, the cells were in situ postfixed with 1% OsO_4_ and 1.5% K_3_Fe(CN)_6_, in 0.07 M Na-cacodylate stained *en bloc* with 0.5% uranyl acetate, dehydrated in ethanol and embedded in Epon. Ultrathin sections were cut parallel to the culture flask bottom, and stained with uranyl acetate and lead citrate. Mm were quantified as present in a phagosome, and autophagosome, or an autolysosome, if they were enclosed by a single membrane lining an otherwise ‘empty’ vacuole, or were surrounded together with some cytosol for at least three quarters by a double membrane lining, or were contained together with irregular vesicles and membrane sheets by a single membrane, respectively.

### *In vitro* ubiquitin reactions & FACS

Cell free ubiquitination reactions were performed by adaptation of previously described methods [48]. After preparation as described above for macrophage infection, approximately 10^9^ Mm were added to 200 µg S-100 HeLa cell lysate, 5 mM MG-132, 4 µM ubiquitin aldehyde to inhibit deubiquitinases, 5 µL of energy regenerating system including phosphocreatine and phosphocreatine kinase and 600 µM ubiquitin (Boston Biochem, Cambridge, MA). After 2 hours shaking at 500 rpm at 36°C, Mm were fixed with 4% PFA in PBS, and then stained with FK2 or anti-gp120 isotype control, followed by APC-conjugated goat anti mouse IgG (Jackson ImmunoResearch, West Grove, PA). To test whether urea could disrupt ubiquitin association, WT Mm were washed twice before PFA fixation with PBS or 8 M urea, fixed and stained as described above. Flow cytometry was performed on stained bacteria using a FACSCaliber (BD Biosciences, San Jose, CA), collecting between 10,000 and 100,000 events per sample. Data analysis was performed using FlowJo Flow Cytometry Analysis Software (Tree Star, Ashland, Oregon). The relative mean fluorescence intensity (MFI) for each sample was determined by dividing the MFI of the FK2- stained sample by the MFI of a ubiquitinated WT Mm sample stained with the anti-gp120 isotype control.

### Fluorescein labeling of Mm

The surface of Mm was labeled with 6-(fluorescein-5-(and-6)-carboxyamido)hexanoic acid, succinimidyl ester 5 (Invitrogen, Carlsbad, CA) as previously described [30].

### Staining with K48- and K63-linkage–specific polyubiquitin antibodies

Infected macrophages were stained with monoclonal antibodies recognizing K48-linked polyubiquitin or K63-linked polyubiquitin [26] after fixation and permeabilization with 0.1% saponin. In some samples, antibodies were incubated at room temperature for 1 h with K48 or K63 tetraubiquitin chains in 10-fold excess (Boston Biochem, Cambridge, MA) prior to addition to the infected macrophages, as previously described [26]. Four independent competitions were performed in parallel. Bound antibody was visualized with goat anti-human IgG-Alexa 594 (Invitrogen, Carlsbad, CA). Comparison of the percent of Mm stained with K48 and K63 was made using a chi-square test for equality of proportions.

### Induction of autophagy

Macrophages were washed with PBS and incubated for 2 hours at 32°C with either EBSS (Sigma Aldrich, St. Louis, MO) or macrophage growth media containing 3% FBS & 3% CMG14-12 supernatant with 50 µg/mL rapamycin (Calbiochem, San Diego, CA) added. As controls, macrophages were incubated in identically constituted growth media without rapamycin, or in full growth media containing 10% FBS & 10% CMG14-12. Cells were then infected and incubated as described for 4 and 24 hours. For immunofluorescence, cells were permabilized with 0.1% saponin for 6 minutes and stained with anti-LC3 followed by goat anti rabbit IgG- F(ab')_2_ (Invitrogen, Carlsbad, CA). Murine IFN-γ was from Sigma Aldrich (St. Louis, MO).

### Immunoblot analysis of LC3

Detection of LC3-I and LC3-II was performed essentially as previously described [49]. Briefly, after cell lysis, protein separation by SDS-PAGE, and transfer, blots were incubated with anti-LC3 overnight at 4°C. Bound LC3 antibody was detected with goat anti-rabbit conjugated to horse radish peroxidase (Jackson ImmunoResearch, West Grove, PA). Blots were stripped and reprobed for GAPDH as a loading control. Chemiluminescence was quantitated with a ChemiDoc XRS from and quantified using Quantity One Analysis Software (Bio-Rad Laboratories, Hercules, CA) by dividing the adjusted intensities for each sample to the adjusted intensity for GAPDH.

## Supporting Information

Figure S1 Differential permeabilization of macrophage membranes by digitonin and electron microscopy quantitation of vacuolar association of phagocytosed Mm. (A) Macrophages were incubated with 4 µm latex beads carrying biotinylated BSA at 32 degrees for 2 hours to mimic conditions of Mm infection. Following permeabilization with digitonin (top row) or with digitonin and Triton X-100 (bottom row), macrophages were incubated with streptavidin-Alexa 594 as described in Materials and Methods. Fluorescence (left panels) and phase (right panels) images of microscopic fields were compared to determine the percentage of latex beads that stained with streptavidin after each permeabilization condition. Note that the one latex bead stained after digitonin alone (lower right) appears to be at the edge of a cell and the fluorescent particle in the upper left is not bead-associated. (B) The percentage of ΔRD1 (n = 85) and WT (n = 177) Mm in the cytoplasm, outside of a phagosomal membrane, at 3.5 HPI was determined by transmission EM. (C) Electron micrograph ΔRD1 Mm surrounded by phagosome membranes (arrows) at 3.5 HPI. Scale bar, 500 nm.
(4.25 MB TIF)

Figure S2 Ubiquitin chain specificity of K48 and K63 antibodies and differential permeabilization showing cytoplasmic localization of ubiquitinated Mm at 3.5 HPI. (A) Macrophages were infected with GFP-expressing WT Mm, permeabilized, and stained with anti-K48 ubiquitin chains (top row) or anti-K63 ubiquitin chains (bottom row) in red. Specificity of K48 and K63 antibodies was tested by competition with K48 and K63 tetraubiquitin chains as described in Materials and Methods. Shown are representative images of staining with each antibody after addition of no competitor (left), K48 (middle), or K63 chains (right). No ubiquitin chain-specific staining of bacteria was detected when the appropriate competitor was used. (B) Macrophages infected with WT Mm expressing GFP were stained with anti-Mm antibody (red) and FK2 (blue) 3.5 HPI after permeabilization with digitonin to detect only cytoplasmic Mm. Merged image from the green, red, and blue panels is shown on the right. This image shows 100% phagosomal escape, which is not representative of the whole population of Mm at this time, but rather depicts several examples of detection of ubiquitinated Mm in the cytoplasm.
(3.02 MB TIF)

Figure S3 Ubiquitin association of iipA mutant and urea washing to remove noncovalently attached ubiquitin from Mm. (A) Macrophages were infected with *iipA* mutant Mm [27] and ubiquitination of the bacteria was determined as described in Materials and Methods 48 HPI. Arrows point to several ubiquitinated Mm. (B) Following ubiquitination *in vitro* as described in Materials and Methods, WT Mm were washed twice with PBS (for isotype control and WT) or with 8 M urea (WT+urea), and bound ubiquitin quantitated using flow cytometry after staining with the isotype control anti-gp120 or FK2 for WT and WT+urea. Graph shows the mean fluorescence intensity divided by the mean fluorescence intensity of a ubiquitinated WT sample stained with isotype control in two independent experiments.
(1.25 MB TIF)

Figure S4 LC3 staining and autophagy induction in macrophages. (A) Macrophages were infected with GFP-expressing WT Mm for 24 hours, permeabilized with saponin, and stained for LC3 (left panel) and FK2 (middle panel). Right panel shows a merge of LC3, FK2, and Mm-GFP; LC3 associates with a Mm that is not ubiquitinated (arrow). (B) Macrophages were incubated for 2 hours with EBSS or rapamycin (50 µg/mL) and then infected with GFP-expressing WT Mm in the same media. 24 HPI, infected macrophages were stained for LC3 (red) and FK2 (blue). Merged images of all fluorescence channels are shown for EBSS (left) and rapamycin (right). Although there is increased aggregation of LC3 in response to the autophagic stimuli (compare with LC3 staining in (A)), there is minimal association of LC3 with ubiquitinated bacteria (arrows) (Scale bars, 10 µm). (C) LC3 association with Mm was quantitated for all intracellular Mm (diamonds, blue line) and for ubiquitinated Mm (squares, red line) 4 and 24 HPI. Data are graphed as mean±SD for three independent experiments. (D) Macrophages were incubated for 2 hours with media or 300 U/ml IFN-γ and infected with WT Mm for 4 or 24 HPI, fixed and stained for LAMP-1 and FK2. Graph shows the percent ubiquitinated Mm associated with LAMP-1 in at least fifty macrophages, ±SD for three independent experiments for 4 HPI and two independent experiments for 24 HPI. (E) 2, 6, and 24 h after infection with WT Mm, macrophages were lysed and LC3-II quantitated as described in Materials and Methods, as the relative level of LC3-II of infected macrophages (triangles, green line) or macrophages incubated with EBSS (squares, red line) compared to the uninfected control incubated in 3% FCS and 3% CMG (diamonds, blue line), normalized to the level of GAPDH intensity for each condition. Mm infection did not affect the low level of LC3-II present at any time point. Average of two experiments is shown. (F) Macrophages were incubated in media containing 10% FCS and 10% CMG supernatant (2nd column); media containing 3% FCS and 3% CMG (third column); rapamycin (4th column), or EBSS (5th column) for 2 h. The 10% FCS/10% CMG condition was included to show that the 3% FCS/3% CMG media used during Mm infection does not induce autophagy. Cell lysate was separated by SDS-PAGE and LC3 detected by WB, and the levels of LC3-II normalized to GAPDH levels, quantitated as described in Materials and Methods. The graph depicts the average of the intensity of the normalized LC3-II band for each condition divided by the normalized LC3-II band from macrophages taken directly from culture at the start of the experiment (Time 0) for three independent experiments. The Western Blot is from a representative experiment. (G) ATG5+/+ and ATG5−/− MEFs were starved in EBSS for 4 h and LC3-II levels were compared by Western blot.
(2.24 MB TIF)

Figure S5 Association of host-derived membranes with ubiquitinated shed Mm cell wall molecules. Macrophages were preincubated with Cholera Toxin B-Alexa 594 (red) for 8 minutes and infected with WT for 3.5 hours. Cells were then fixed and stained with FK2 (blue). Purple shown in the merge are areas of overlap between red and blue.
(2.63 MB TIF)
