# Supplementary figures and images for: Atg5-Independent Sequestration of Ubiquitinated Mycobacteria
Source: PLoS Pathog. 2009 May 15;5(5):e1000430. doi: 10.1371/journal.ppat.1000430 (PMC2673685; doi:10.1371/journal.ppat.1000430)

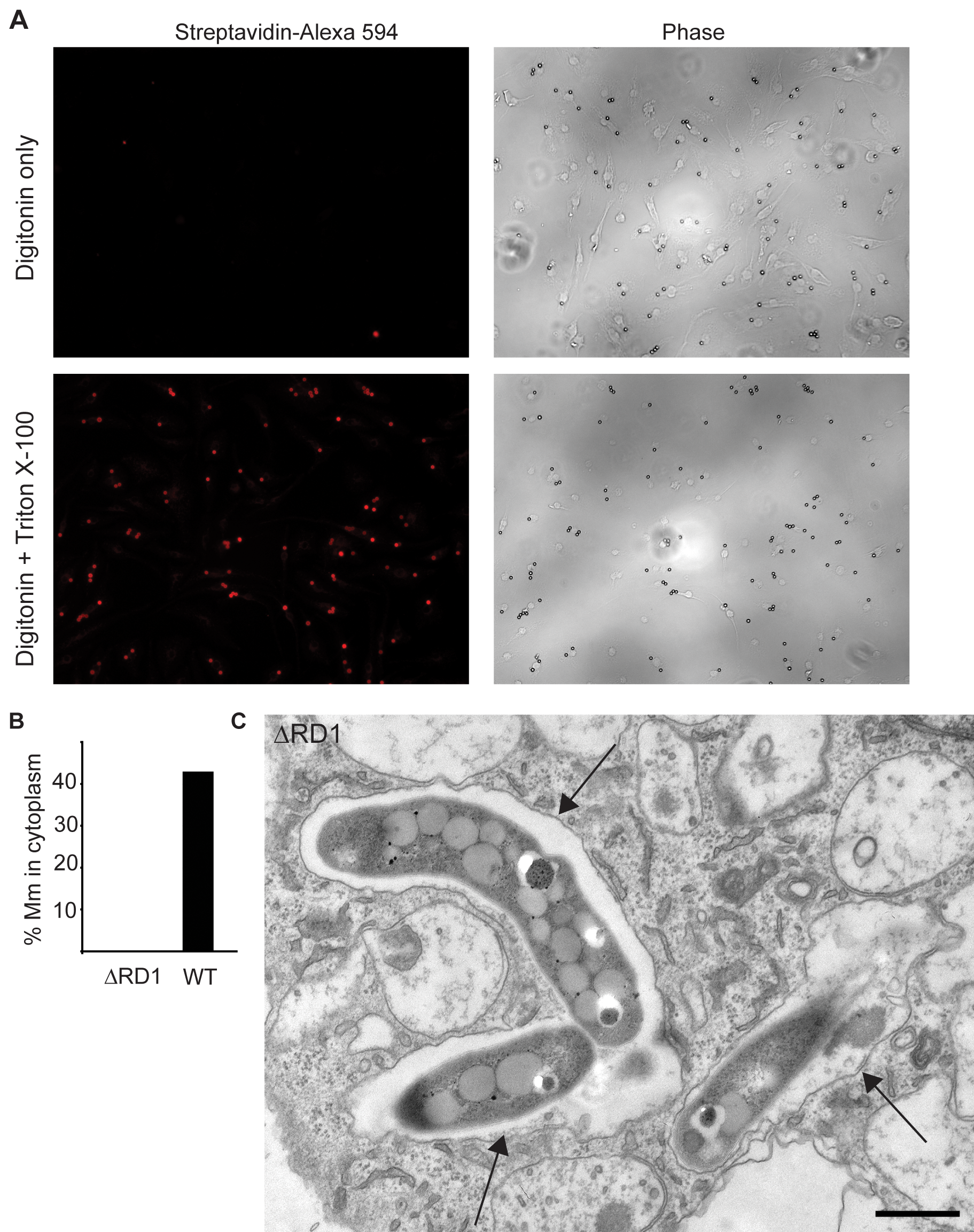

Supplement: Figure S1 — Differential permeabilization of macrophage membranes by digitonin and electron microscopy quantitation of vacuolar association of phagocytosed Mm. (A) Macrophages were incubated with 4 µm latex beads carrying biotinylated BSA at 32 degrees for 2 hours to mimic conditions of Mm infection. Following permeabilization with digitonin (top row) or with digitonin and Triton X-100 (bottom row), macrophages were incubated with streptavidin-Alexa 594 as described in Materials and Methods. Fluorescence (left panels) and phase (right panels) images of microscopic fields were compared to determine the percentage of latex beads that stained with streptavidin after each permeabilization condition. Note that the one latex bead stained after digitonin alone (lower right) appears to be at the edge of a cell and the fluorescent particle in the upper left is not bead-associated. (B) The percentage of ΔRD1 (n = 85) and WT (n = 177) Mm in the cytoplasm, outside of a phagosomal membrane, at 3.5 HPI was determined by transmission EM. (C) Electron micrograph ΔRD1 Mm surrounded by phagosome membranes (arrows) at 3.5 HPI. Scale bar, 500 nm. (4.25 MB TIF) [file ppat.1000430.s001.tif]

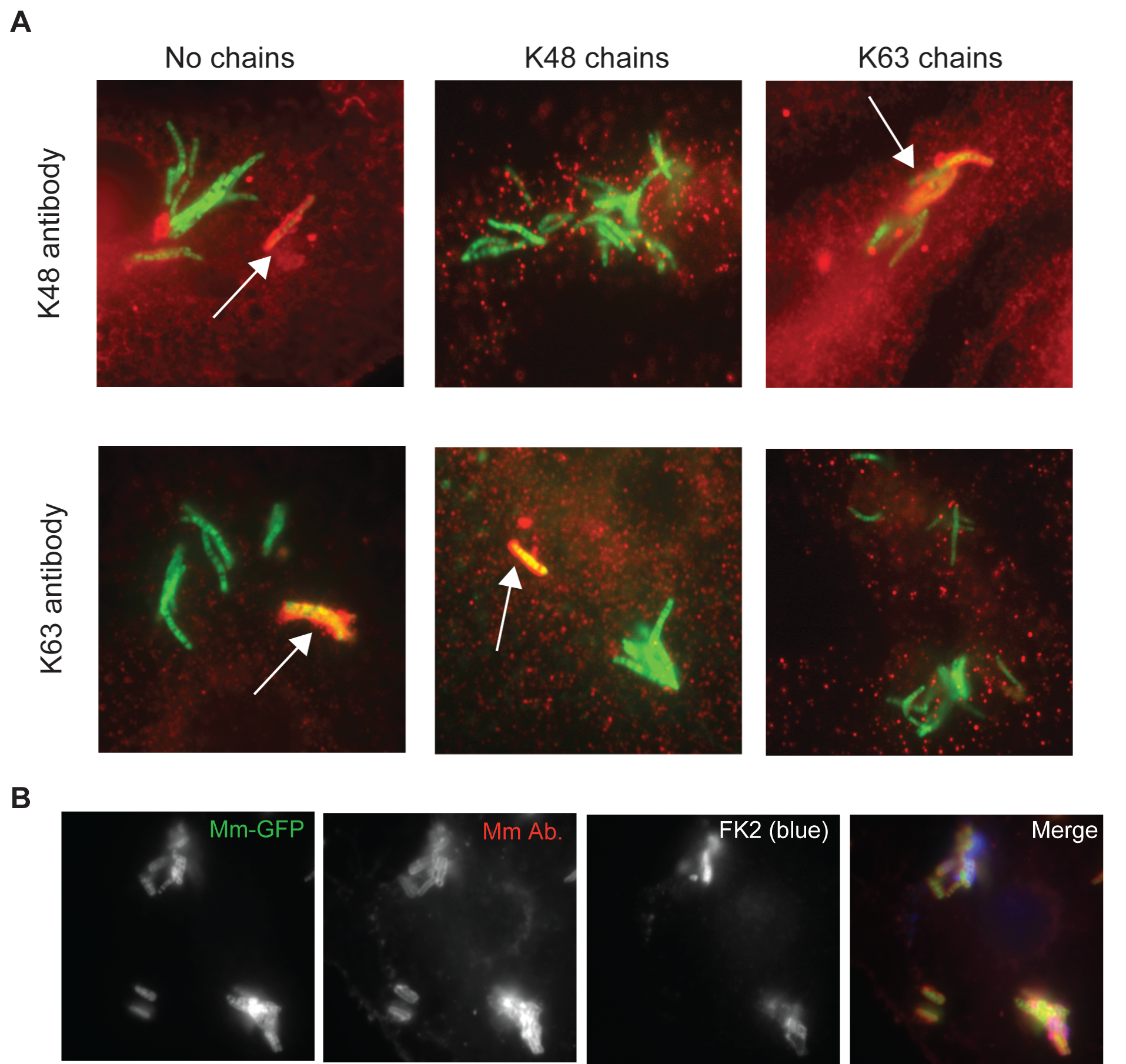

Supplement: Figure S2 — Ubiquitin chain specificity of K48 and K63 antibodies and differential permeabilization showing cytoplasmic localization of ubiquitinated Mm at 3.5 HPI. (A) Macrophages were infected with GFP-expressing WT Mm, permeabilized, and stained with anti-K48 ubiquitin chains (top row) or anti-K63 ubiquitin chains (bottom row) in red. Specificity of K48 and K63 antibodies was tested by competition with K48 and K63 tetraubiquitin chains as described in Materials and Methods. Shown are representative images of staining with each antibody after addition of no competitor (left), K48 (middle), or K63 chains (right). No ubiquitin chain-specific staining of bacteria was detected when the appropriate competitor was used. (B) Macrophages infected with WT Mm expressing GFP were stained with anti-Mm antibody (red) and FK2 (blue) 3.5 HPI after permeabilization with digitonin to detect only cytoplasmic Mm. Merged image from the green, red, and blue panels is shown on the right. This image shows 100% phagosomal escape, which is not representative of the whole population of Mm at this time, but rather depicts several examples of detection of ubiquitinated Mm in the cytoplasm. (3.02 MB TIF) [file ppat.1000430.s002.tif]

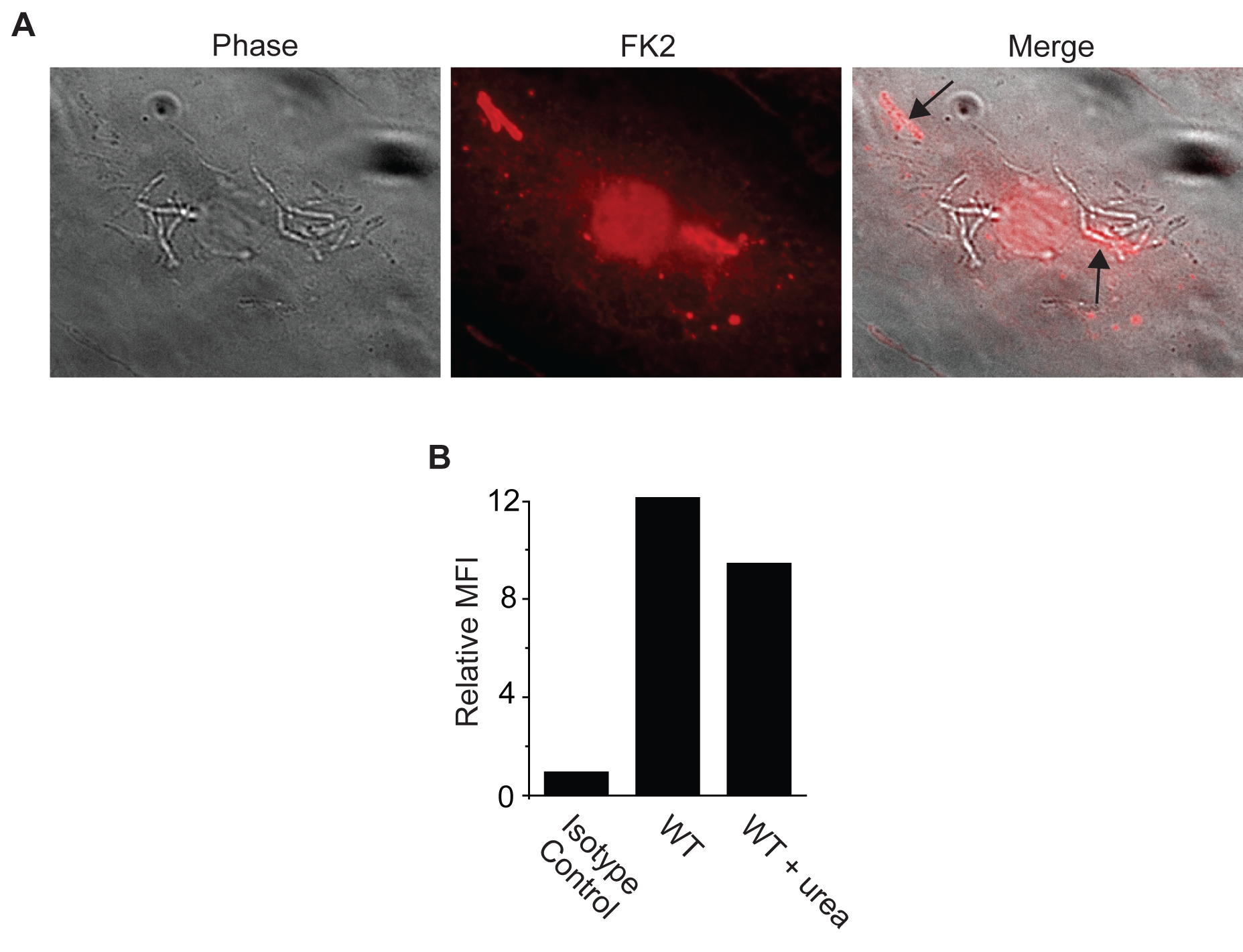

Supplement: Figure S3 — Ubiquitin association of iipA mutant and urea washing to remove noncovalently attached ubiquitin from Mm. (A) Macrophages were infected with iipA mutant Mm [27] and ubiquitination of the bacteria was determined as described in Materials and Methods 48 HPI. Arrows point to several ubiquitinated Mm. (B) Following ubiquitination in vitro as described in Materials and Methods, WT Mm were washed twice with PBS (for isotype control and WT) or with 8 M urea (WT+urea), and bound ubiquitin quantitated using flow cytometry after staining with the isotype control anti-gp120 or FK2 for WT and WT+urea. Graph shows the mean fluorescence intensity divided by the mean fluorescence intensity of a ubiquitinated WT sample stained with isotype control in two independent experiments. (1.25 MB TIF) [file ppat.1000430.s003.tif]

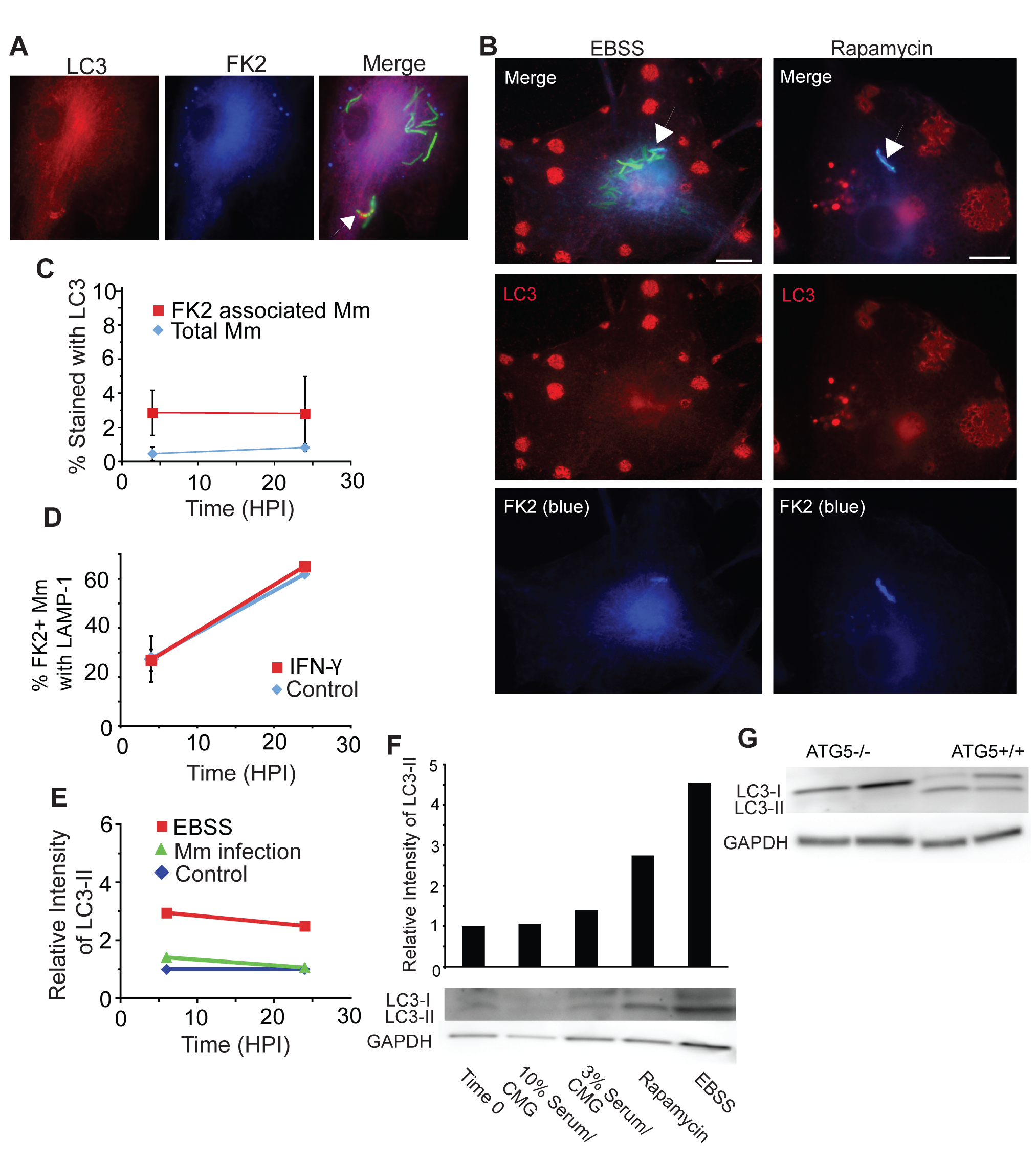

Supplement: Figure S4 — LC3 staining and autophagy induction in macrophages. (A) Macrophages were infected with GFP-expressing WT Mm for 24 hours, permeabilized with saponin, and stained for LC3 (left panel) and FK2 (middle panel). Right panel shows a merge of LC3, FK2, and Mm-GFP; LC3 associates with a Mm that is not ubiquitinated (arrow). (B) Macrophages were incubated for 2 hours with EBSS or rapamycin (50 µg/mL) and then infected with GFP-expressing WT Mm in the same media. 24 HPI, infected macrophages were stained for LC3 (red) and FK2 (blue). Merged images of all fluorescence channels are shown for EBSS (left) and rapamycin (right). Although there is increased aggregation of LC3 in response to the autophagic stimuli (compare with LC3 staining in (A)), there is minimal association of LC3 with ubiquitinated bacteria (arrows) (Scale bars, 10 µm). (C) LC3 association with Mm was quantitated for all intracellular Mm (diamonds, blue line) and for ubiquitinated Mm (squares, red line) 4 and 24 HPI. Data are graphed as mean±SD for three independent experiments. (D) Macrophages were incubated for 2 hours with media or 300 U/ml IFN-γ and infected with WT Mm for 4 or 24 HPI, fixed and stained for LAMP-1 and FK2. Graph shows the percent ubiquitinated Mm associated with LAMP-1 in at least fifty macrophages, ±SD for three independent experiments for 4 HPI and two independent experiments for 24 HPI. (E) 2, 6, and 24 h after infection with WT Mm, macrophages were lysed and LC3-II quantitated as described in Materials and Methods, as the relative level of LC3-II of infected macrophages (triangles, green line) or macrophages incubated with EBSS (squares, red line) compared to the uninfected control incubated in 3% FCS and 3% CMG (diamonds, blue line), normalized to the level of GAPDH intensity for each condition. Mm infection did not affect the low level of LC3-II present at any time point. Average of two experiments is shown. (F) Macrophages were incubated in media containing 10% FCS and 10% CMG super [file ppat.1000430.s004.tif]

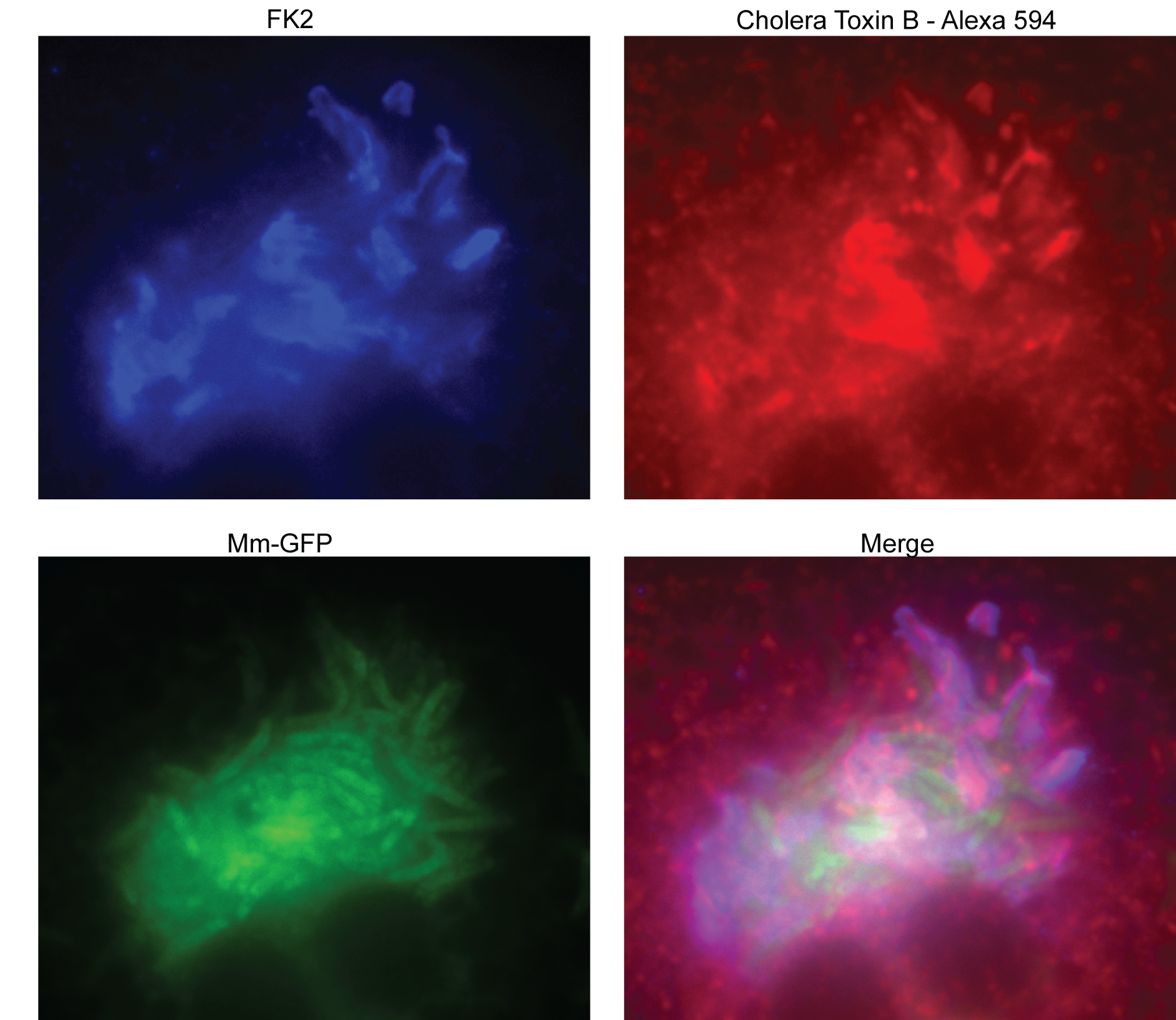

Supplement: Figure S5 — Association of host-derived membranes with ubiquitinated shed Mm cell wall molecules. Macrophages were preincubated with Cholera Toxin B-Alexa 594 (red) for 8 minutes and infected with WT for 3.5 hours. Cells were then fixed and stained with FK2 (blue). Purple shown in the merge are areas of overlap between red and blue. (2.63 MB TIF) [file ppat.1000430.s005.tif]
